# Effect of pretreatment strategies on halophyte *Atriplex crassifolia* to improve saccharification using thermostable cellulases

**DOI:** 10.3389/fbioe.2023.1135424

**Published:** 2023-02-21

**Authors:** Ali Nawaz, Khadija Qadoos, Ikram Ul Haq, Yiwei Feng, Hamid Mukhtar, Rong Huang, Kankan Jiang

**Affiliations:** ^1^ School of Basic Medical Sciences and Forensic Medicine, Hangzhou Medical College, Hangzhou, China; ^2^ Institute of Industrial Biotechnology, Government College University, Lahore, Pakistan; ^3^ School of Clinical Medicine, Hangzhou Medical College, Hangzhou, China

**Keywords:** biorefinery, bioethanol, halophytes, pre-treatment, delignification, enzymatic hydrolysis, thermostable cellulases

## Abstract

Bioethanol is believed to be an influential revolutionary gift of biotechnology, owing to its elevating global demand and massive production. Pakistan is home to a rich diversity of halophytic flora, convertible into bounteous volumes of bioethanol. On the other hand, the accessibility to the cellulosic part of biomass is a major bottleneck in the successful application of biorefinery processes. The most common pre-treatment procedures existent include physicochemical and chemical approaches, which are not environmentally benign. To overcome these problems, biological pre-treatment has gained importance but the drawback is the low yield of the extracted monosaccharides. The current research was aimed at exploring the best pre-treatment method for the bioconversion of halophyte *Atriplex crassifolia* into saccharides using three thermostable cellulases. *Atriplex crassifolia* was subjected to acid, alkali and microwave pre-treatments, followed by compositional analysis of the pre-treated substrates. Maximum delignification i.e. 56.6% was observed in the substrate pre-treated using 3% HCl. Enzymatic saccharification using thermostable cellulases also validated the results where the highest saccharification yield i.e. 39.5% was observed for the sample pre-treated using same. Maximum enzymatic hydrolysis of 52.7% was obtained for 0.40 g of the pre-treated halophyte *Atriplex crassifolia* where Endo-1,4- β -glucanase (300U), Exo-1,4- β -glucanase (400U) and β -1,4-glucosidase (1000U) were simultaneously added and incubated for 6 h at 75°C. The reducing sugar slurry obtained after optimization of saccharification was utilized as glucose in submerged fermentation for bioethanol production. The fermentation medium was inoculated with *Saccharomyces cerevisiae,* incubated at 30°C and 180 rpm for 96 h. Ethanol production was estimated using potassium dichromate method. Maximum production of bioethanol i.e. 16.33% was noted at 72 h. It can be concluded from the study that *Atriplex crassifolia* owing to its high cellulosic content after pre-treatment using dilute acid method, yields substantial amount of reducing sugars and high saccharification rates when subjected to enzymatic hydrolysis using thermostable cellulases, under optimized reaction conditions. Hence, the halophyte *Atriplex crassifolia* is a beneficial substrate that can be utilized to extract fermentable saccharides for bioethanol production.

## 1 Introduction

Fossil-based fuels are not only non-renewable energy resources having detrimental impact on the environment, but their limited availability in depleting reservoirs is not sufficient to fulfill the global energy needs (Li et al., 2020; Vu et al., 2020). Renewable energy comes along sustainable development, energy security and economic growth (Den et al., 2018). Biorefinery is based on the sustainable conversion and efficient valorization of raw material (lignocellulosic biomass, waste, etc.) into wide range of bio-based energy products (Hingsamer and Jungmeier, 2019).

Bioethanol fuel is believed to be an influential revolutionary gift of biotechnology, owing to its elevating global demand and massive production. Biofuels, based on their source of biomass have been categorized into four generations. First-generation biofuels are a product of edible crops, for instance, wheat and corn (Nanda et al., 2018). Second-generation biofuels are a product of non-edible crops; this includes the waste of food crops, agricultural and forest residues, chips of wood, and waste from cooking oil (Nanda et al., 2018). In the modern world of today where overpopulation and poverty are major concerns, employing first generation biofuels is not that favorable, keeping in view the scarcity of available food, fresh water, and arable land resources. Whereas, the second-generation biofuel energy crops can be easily produced on degraded saline lands using scarce water resources to minimize the competition with food production (Moioli et al., 2018).

It is true that 70% of the earth is made of water; on the other hand, a bulk amount of this percentage remains unsuitable for irrigational purposes and does not support human intake, owing to high salinity rates. Moreover, the limited availability of arable land is also subject to increasing salinity, because of continuous irrigation, (that adds to the existing salts); this remains a major challenge to enhance agricultural practices (Flowers and Colmer, 2008). Precise and recent estimates on the global extent of salinity-afflicted land is unavailable, data is variable according to different information sources (Shahid et al., 2018). However, a global figure of 952.2 million ha has been reported (Arora et al., 2016). Evidently, the utilization of this massive chunk of space to grow and harvest biofuel feedstocks, which can be subjected to irrigation using seawater, can help eliminate the major barrier linked to farming for biofuel production (Brown, 2019).

Halophytes, which are capable of growth in salinity-afflicted soils are inexpensive sources of lignocellulosic biomass (Joshi et al., 2020). Halophytes produce oilseeds and lignocellulosic biomass; both the products can be exploited for biofuel production. Among the topmost promising genera exist *Salicornia* (glasswort), *Suaeda* (sea-blite), *Atriplex* (saltbush), *Distichlis* (saltgrass) and *Batis* (saltwort) (Abideen et al., 2011). Cultivation of halophytes in saline-afflicted regions will spare fresh water resources and arable agricultural soils for food, and provide lignocellulosic feedstock of desirable quality for conversion into biofuel (Yuan et al., 2011).

The genus *Atriplex* belongs to the family Amaranthaceae and is home to approximately 300 saltbush species. Saltbush species are resistant towards adaptation; majority of them have their habitats in semi-arid and arid areas globally (Paneque, 2018). Species named *Atriplex crassifolia*, a wild, annual halophyte native to semi-arid and arid areas of Punjab, was the subject of study in this research.

Pre-treatment of lignocellulosic biomass is a basic prerequisite for effective enzymatic hydrolysis of biomass. Pre-treatment ensures disruption of the complicated network of lignocellulose and ensures sustainable production of valuable products by enhancing the surface area of biomass, so that the cellulases can easily act upon the cellulosic content (Meng et al., 2020; Padilla-Rascón et al., 2020). The most common pre-treatment procedures existent include physicochemical and chemical approaches, which are not environmentally benign. To overcome these problems, biological pre-treatment has gained importance but the drawback is the low yield of the extracted monosaccharides (Rezania et al., 2020). Furthermore, different green processes have evolved for the pre-treatment of biomass (Lyu et al., 2019). Although, these techniques offer promising benefits, their wide-scale applicability at industrial level demands high capital cost (Bhatia et al., 2020; Shen et al., 2020). Therefore, extensive research is a requirement to develop the most applicable technique for pre-treatment that ensures ease of applicability along with affordability.

Alkaline solutions have been long used for the pre-treatment of lignocellulosic biomass. The complex structure of lignin composed of alkyl-aryl linkages are subjected to disruption utilizing alkalis such as ammonium hydroxide, sodium hydroxide, calcium hydroxide and lime (Chen et al., 2013). The use of acids for pre-treatment is another chemical pre-treatment technique used to degrade the lignocellulosic biomass, as it cleaves the glycosidic bonds present within the biomass structure (Sahoo et al., 2018). Nitric acid, phosphoric acid, sulfuric acid and hydrochloric acid are various examples of the acids that have been analyzed for their capability to pre-treat lignocellulosic biomass, even at industrial levels (Solarte-Toro et al., 2019).

Pre-treatment using microwave is another technique to disrupt the construction of lignocellulosic biomass. Irradiation of the microwave causes acceleration of chemical reactions within the biomass. The vibrations created by the heat of microwave, generate hot spots within the structure of biomass (Hassan et al., 2018). Microwave irradiation for pre-treatment of biomass is a favorable method in the domain of biomass biorefinery, owing to the heat that not affects the surface of biomass but also elevates the temperature inside the structure of biomass, being more energetic and effective compared to conventional heating techniques (Pellera and Gidarakos, 2017).

Saccharification is the process where complex carbohydrates are broken down into simple sugars. Following the pre-treatment of lignocellulosic biomass, saccharification is the next significant step in the bioconversion of cellulosic content into monosaccharides (Kucharska et al., 2020). Enzymatic saccharification is generally conducted employing cellulases and hemicellulases (Kumar and Sharma, 2017). Cellulase is not a term used to define a single enzyme, infact it is a complex of multiple enzymes (Thapa et al., 2020). Generally, the term cellulases is used to denote three glycoside hydrolases i.e., (endocellulases, exocellulases and β -glucosidases), which play role in the bioconversion of cellulosic content within biomass to reducing sugars (Champreda et al., 2019). Hydrolysis efficiency depends upon different physicochemical parameters which include incubation pH, time, temperature, agitation speed, particle size, enzyme/substrate ratio, etc. (Chavan and Gaikwad, 2021; Faizal et al., 2021).

Fermentation is an important step in bioethanol production, which can be conducted either separately or in combination with saccharification. In this research, separate hydrolysis and fermentation (SHF) has been conducted, as it is the most favorable technique concerning bioethanol production. It facilitates self-regulating optimization of saccharification for maximizing sugar extraction and that of fermentation for ethanol production (Moodley and Kana, 2019).

Pakistan is home to a rich diversity of halophytic flora, convertible into bounteous volumes of bioethanol. The current research, utilizing halophytes, aimed to provide promising results in developing sustainable bioethanol fuel for the citizens, which is also a foremost need of the country, keeping in view the ever-increasing hike in petroleum prices, climatic vulnerability, decreasing arable land, high salinity, scarce fresh water resources, food insecurity, overpopulation, energy demand and poverty.

## 2 Materials and methods

### 2.1 Chemicals

All chemicals used in the present study were of analytical grade and purchased from authentic suppliers of Sigma, Daejung, Acros Organics and Merck Ltd.

#### 2.1.1 Thermophilic cellulases

The genetically engineered thermophilic cellulases were obtained from the project entitled “Production of bioenergy from plant biomass” at Institute of Industrial Biotechnology, GC University Lahore, Pakistan.

#### 2.1.2 Substrate

The halophyte *Atriplex crassifolia* was collected in early January 2021 from the fields of KSK campus, GCU Lahore, Punjab Pakistan. The biomass was grinded into fine powder and dried in hot air oven at 105°C for 2 h. The dried biomass was sieved and stored in labelled zip-lock bags at room temperature for further use.

### 2.2 Reagent preparation and pre-treatment

#### 2.2.1 Alkali pre-treatment

Alkali pre-treatment of *Atriplex crassifolia* was out using different concentrations of NaOH (1%, 2%, 3%, 4% and 5%) as the pre-treatment reagents. Oven dried substrates were taken in an amount of 5 g and mixed in 50 mL of NaOH solution with varying concentrations, using 100 mL air tight reagent bottles. The screw capped reagent bottles were subjected to a temperature of 121°C for a time period of 60 min in an autoclave. Pre-treated substrates were filtered and washed by distilled water twice to eliminate attached alkali and other components produced during the pre-treatment. The substrates were air-dried. The dried substrates were put in sterilized polythene zipper bags for further use (Binod et al., 2011).

#### 2.2.2 Acid pre-treatment

Acid pre-treatment of *Atriplex crassifolia* was carried out using different concentrations of HCl (1%, 2%, 3%, 4% and 5%) as the pre-treatment reagents. Oven dried substrates were taken in an amount of 5 g and mixed in 50 mL of HCl solution with varying concentrations, using 100 mL airtight reagent bottles. The screw capped reagent bottles were subjected to a temperature of 121°C for 60 min in an autoclave. Following pre-treatment, the substrates were filtered and washed twice using distilled water to remove any acidic content or other byproducts formed during pre-treatment. The substrates were allowed to air dry. The dried substrates were stored in sterilized polythene zipper bags for further use (Binod et al., 2011).

#### 2.2.3 Microwave pre-treatment

Microwave pre-treatment of *Atriplex crassifolia* was carried out by varying residence times. Substrates (1 g) were weighed and placed in five different airtight reagent bottles of 50 mL capacity, having 10 mL of distilled water each. The respective airtight bottles were subjected to microwave irradiation for residence time of 1 min, 2 min, 3 min, 4 min and 5 min, respectively. Following pre-treatment, the substrates were filtered. Air dried substrates were stored in sterilized zipper bags for further use (Binod et al., 2011).

### 2.3 Biomass compositional analysis

The untreated and pretreated biomass was analysed for lignocellulosic content using Technical Association of Pulp and Paper Industry (TAPPI) standards.

### 2.4 Enzymatic saccharification of pre-treated *Atriplex crassifolia* biomass

The enzymatic saccharification of pre-treated biomass *Atriplex crassifolia* was carried out by adding 0.25 g of pre-treated substrate in a screw-capped reagent bottle. For enzymatic hydrolysis, Endo-1,4- β -glucanase (200 U) was added to both experimental and control (without substrate) reagent bottles. Both the reagent bottles were incubated at 75°C in a shaking water bath with rpm set at 50 for a period of 2 h. Next, Exo-1,4- β -glucanase (400 U) was added to the same reagent bottles and after incubating the mixture for another 2 h, β -1,4-glucosidase (1000 U) was added to both the reagent bottles. The samples (1 mL) were withdrawn after regular intervals of 1 h to note the release of reducing sugars *via* DNS method, using straight line equation obtained from the standard curve. The percentage saccharification was determined by using Vallander and Eriksson (1987) proposed equation;

$$\%\;Saccharification=\frac{R.S\times V\times F1}{M\times F2}\times 100$$

### 2.5 Optimization of physicochemical parameters to enhance enzymatic saccharification

The physicochemical parameters i.e., incubation time, incubation temperature, substrate concentration, concentration of endo-1,4- β -glucanase, exo-1,4- β -glucanase and β -glucosidase affecting the rate of enzymatic hydrolysis were subjected to optimization studies. One variable at a time experimental design was chosen for the optimization of saccharification parameters.

#### 2.5.1 Sequential addition of cellulases

In sequential addition of cellulases, beginning with the addition of Endo-1,4- β -glucanase (200 U) to the screw-capped reagent bottle enclosing the pre-treated substrate i.e., halophyte *Atriplex crassifolia* (0.25 g), the reaction was carried out in a shaking water bath set at 75°C and 50 rpm. After 2 h of incubation time, Exo-1,4- β -glucanase (400 U) was added to the same reagent bottle for another 2 h. Next, the cellulase i.e., β -1,4-glucosidase was added to the same reagent bottle and incubated for a period of 2 h. In this way, in sequential addition of cellulases, each cellulase was added one after the other and each was incubated for a period of 2 h.

#### 2.5.2 Simultaneous addition of cellulases

Whereas, in simultaneous addition of cellulases, all the three cellulases i.e., Endo-1,4- β -glucanase (200 U), Exo-1,4- β -glucanase (400 U) and β -1,4-glucosidase (1000 U) were added altogether to the screw-capped reagent bottle enclosing the pre-treated substrate i.e., halophyte *Atriplex crassifolia* (0.25 g). The saccharification mixture containing cellulases and the substrate was incubated for a period of 6 h, in a shaking water bath set at 75°C and 50 rpm.

### 2.6 Ethanol fermentation

The saccharified slurry was subjected to ethanol fermentation following the method of Nawaz et al. (2022).

### 2.7 Statistical analysis

All the experiment were run in triplicates and subjected to statistical analysis using SPSS version 16.00. Error bars in the figures of results section depicted standard deviation ( $\left.\pm SD\right)$ among the replicates run, varying significantly at *p* < 0.05.

## 3 Results and Discussions

### 3.1 Biomass compositional analysis

**FIGURE 1:**
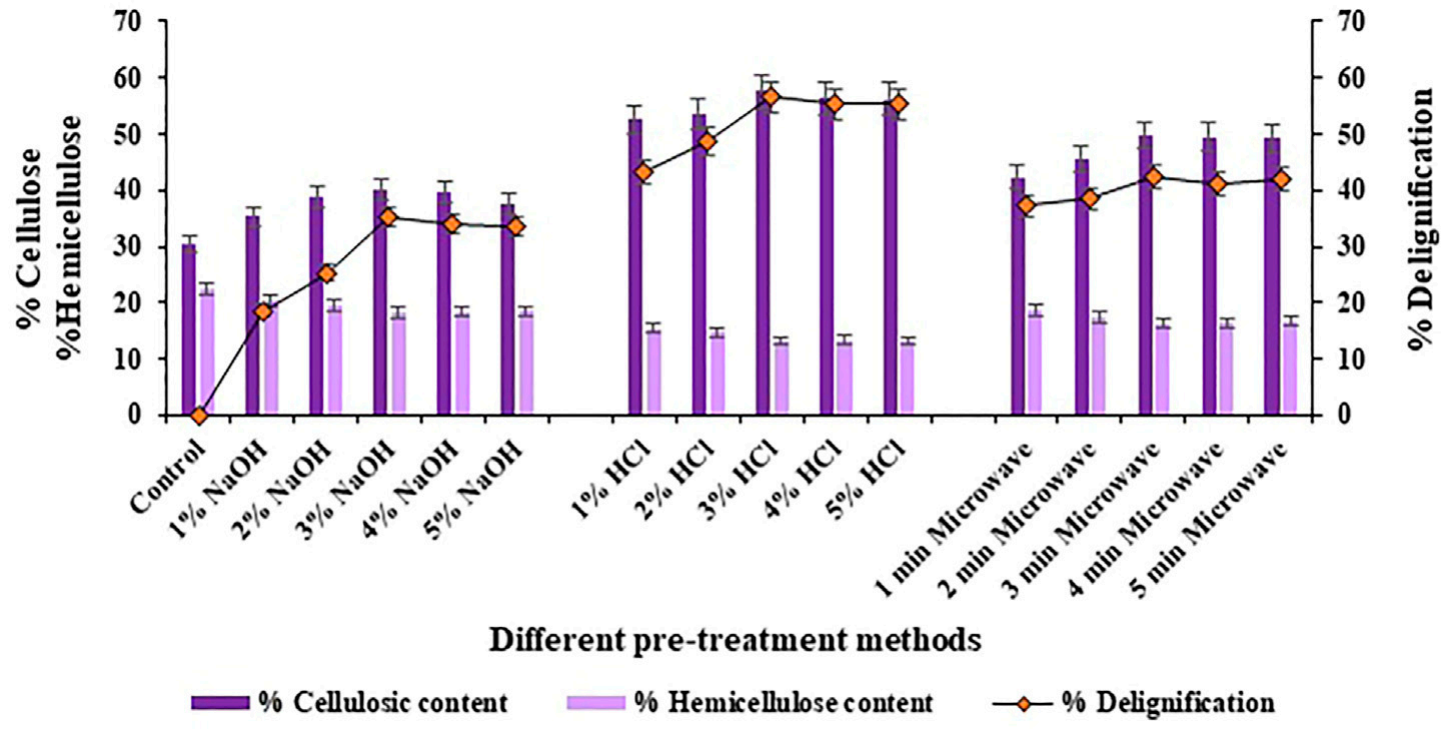
Compositional analysis of halophyte *Atriplex crassifolia* before and after pre-treatment with different methods.

The results of compositional analysis of halophyte *Atriplex crassifolia* after subjecting it to three different pre-treatment techniques revealed that the maximum rate of delignification (56.6%; *p* < 0.05), the maximum cellulosic content (57.7%; *p* < 0.05) and the maximum degradation of hemicellulose was shown by the halophyte pre-treated using 3% hydrochloric acid as the pre-treatment reagent, as shown in Figure 1. Acids when used as pre-treatment reagents are quite competent. Although, they work excellent for the degradation of hemicellulosic content of the biomass, but also contribute well in the removal of lignin, hence enhancing the ease of availability of cellulose for the enzymes, in turn maximizing the release of fermentable saccharides (Sahoo et al., 2018). Few important considerations regarding the associated parameters ensure the appropriate applicability of this method; this includes residence time, pre-treatment temperature, substrate concentration and acid strength. The use of acids maximize the cleaving of the bonds within recalcitrant lignocellulosic structure and ensure effective conversion of the cellulosic material into reducing sugars (Solarte-Toro et al., 2019).

### 3.2 Enzymatic saccharification of pre-treated *Atriplex crassifolia* biomass

**FIGURE 2:**
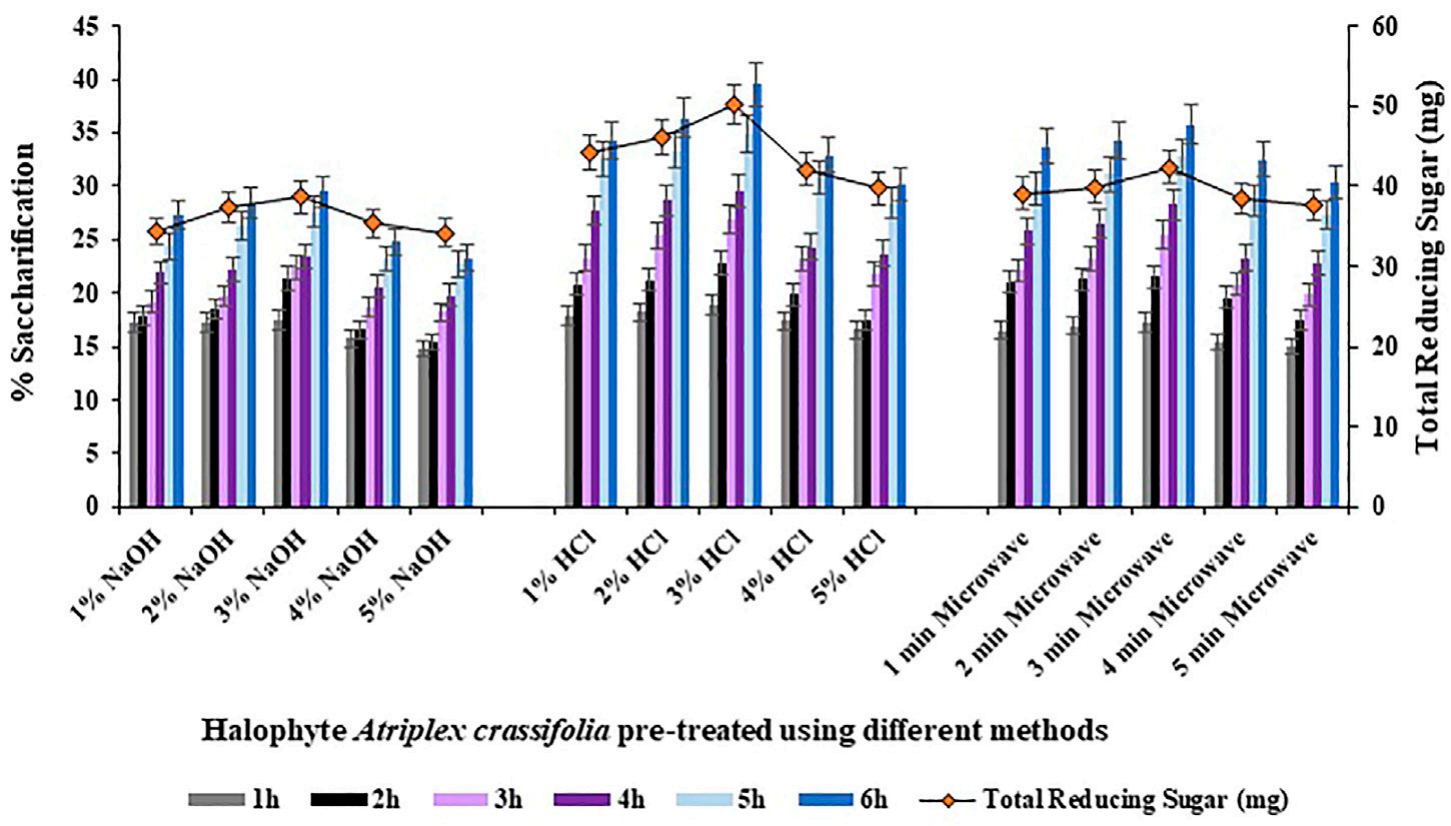
Saccharification studies using halophyte *Atriplex crassifolia* pre-treated with different methods.

Comparison of the overall saccharification yields of the halophyte *Atriplex crassifolia* pre-treated using different methods revealed that the maximum saccharification rate (39.5%; *p* < 0.05) as well as the maximum yield of reducing sugars was shown by the halophyte pre-treated using 3% hydrochloric acid as the pre-treatment reagent, as shown in Figure 2. The results of enzymatic saccharification were parallel to the results of compositional analysis. Therefore, the halophyte *Atriplex crassifolia* pre-treated using 3% hydrochloric acid was chosen for further experiments. The results were in line with those found by Kumar et al. (2020), who worked on varied pre-treatment methods to efficiently convert *Lantana camara* stem into reducing sugars. Of all the pre-treatment methods used, the substrate subjected to pre-treatment using acid in an autoclave, was found to give maximum yield of reducing sugar upon enzymatic hydrolysis. The results were also consistent with Ansari et al. (2021), who screened different halophytes for enzymatic hydrolysis. Results of saccharification revealed that acid pre-treated *P. karka* yielded maximum monosaccharides. Selection of the best pre-treatment method is the key to successful enzymatic hydrolysis, owing to the fact that saccharification of pre-treated substrate is greatly increased when the accessibility and ease of cellulases to the cellulosic fibers is ensured (López-Linares et al., 2015). To carry out pre-treatment of lignocellulosic feedstock, dilute acid method is most frequently studied and is believed to be an economical process for industrial scale production of bioethanol utilizing lignocellulosic biomass (Ruiz et al., 2013).

### 3.3 Optimization of physicochemical parameters affecting saccharification

#### 3.3.1 Effect of cellulases addition method on saccharification

**FIGURE 3:**
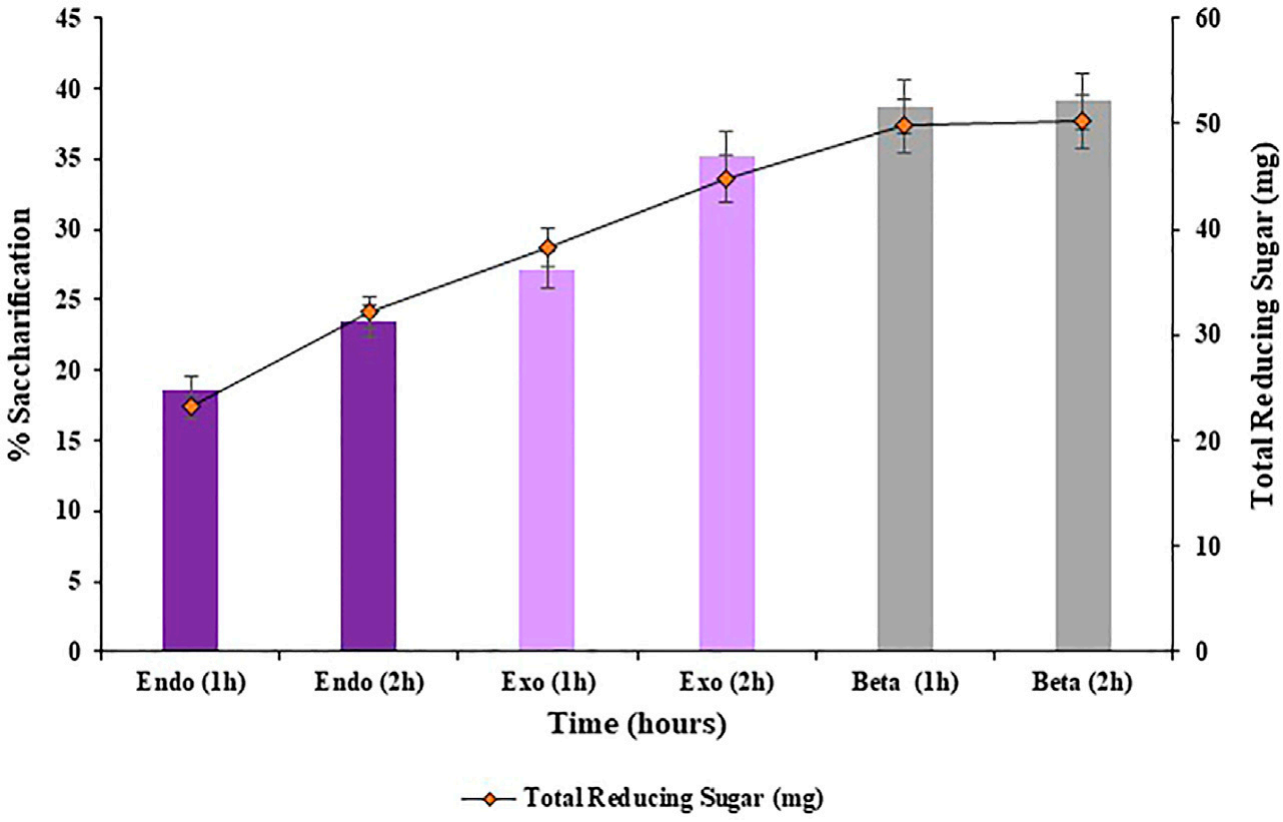
Saccharification of pre-treated halophyte *Atriplex crassifolia* using sequential addition of cellulases.

**FIGURE 4:**
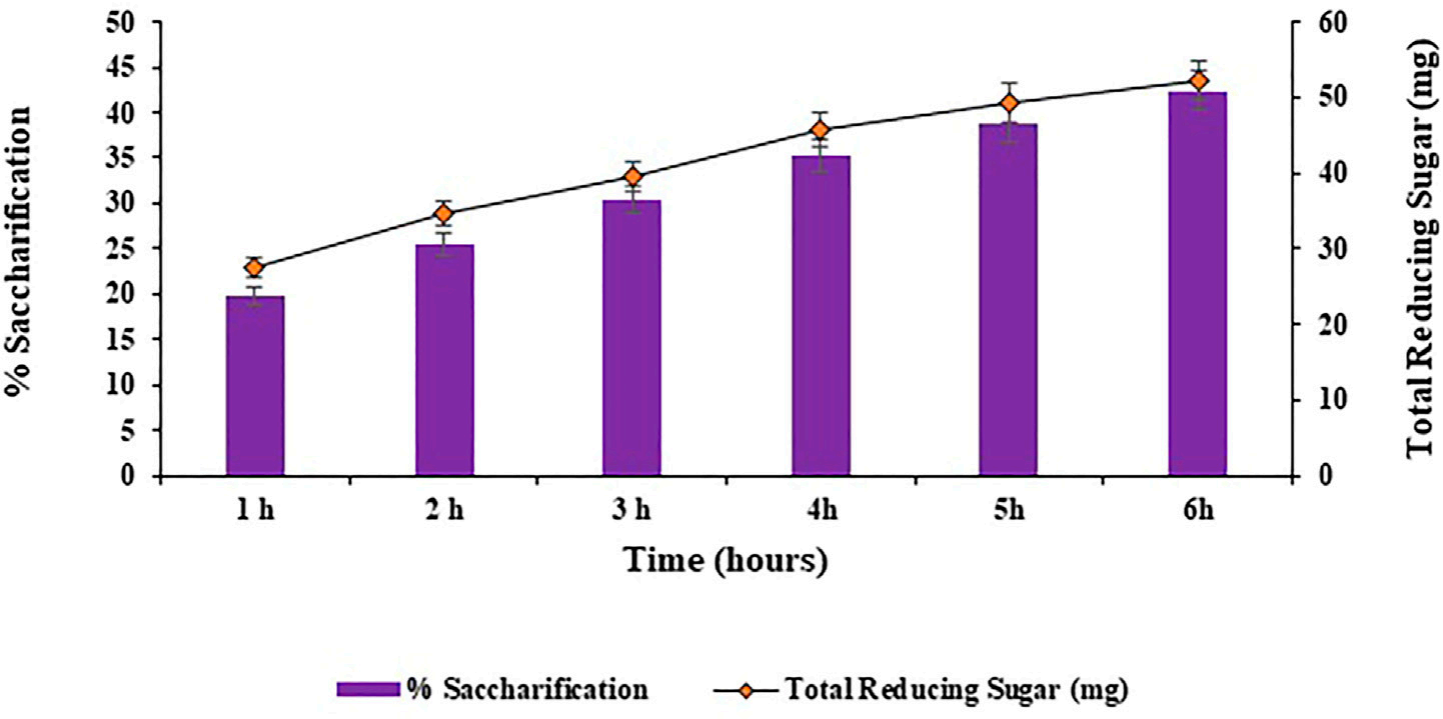
Saccharification of pre-treated halophyte *Atriplex crassifolia* using simultaneous addition of cellulases.

Saccharification was conducted using both, sequential addition of cellulases and by simultaneous addition of cellulases, in order to evaluate the best method of cellulases addition to achieve maximum rate of enzymatic saccharification. Overall, simultaneously adding the cellulases resulted in improved hydrolysis (42.4%; *p* < 0.05) of the substrate i.e., pre-treated halophyte *Atriplex crassifolia* compared to sequential addition of cellulases (39.1%; *p* < 0.05), as shown in Figures 3, 4, respectively. This might be due to the increased activity of cellulases when they act together synergistically, rather than individually. These results were found consistent with Mcintosh and Vancov (2011), who also simultaneously added three enzymes i.e., cellulase, β-glucosidase and xylanase in combination to conduct saccharification of wheat straw, and found favorable results. The synergistic action of enzymes by simultaneous addition of cellulases was found to increase the total sugar release and it was also found to reduce the enzyme loadings 3-fold, making the process economical. Hence, the next optimization reactions of saccharification were conducted using simultaneous method of cellulases addition.

#### 3.3.2 Effect of incubation time on saccharification

**FIGURE 5:**
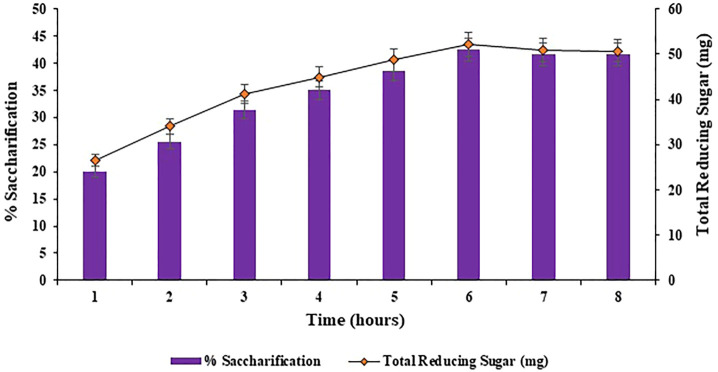
Effect of incubation time on saccharification of pre-treated halophyte *Atriplex crassifolia.*

Different incubation times of 1, 2, 3, 4, 5, 6, 7 and 8 h were analyzed for the bioconversion of substrate i.e., halophyte *Atriplex crassifolia* into saccharides. Maximum bioconversion of (42.5%; *p* < 0.05) was detected after 6 h of incubating the reaction mixture that contained the cellulases and the pre-treated substrate i.e., *Atriplex crassifolia*, as depicted in Figure 5. Increasing the time resulted in a decrease in the yield of saccharification, as evident from Figure 5. It might be because of the reason that with additional rise in incubation time, either the process of product inhibition comes into action due to accretion of glucose and cellobiose, or due to less availability of free cellulose with the passage of time. Results of this research are in accordance with those of Aftab et al. (2017) that highest saccharification of sugarcane bagasse was observed after 6 h of incubation using thermophilic cellulases. These alike results can be attributed to the reason that thermophilic cellulases act more energetically and that when an amalgam of enzymes is employed for saccharification, the overall reaction time for hydrolysis is lessened (Shokrkar and Ebrahimi, 2018).

#### 3.3.3 Effect of incubation temperature on saccharification

**FIGURE 6:**
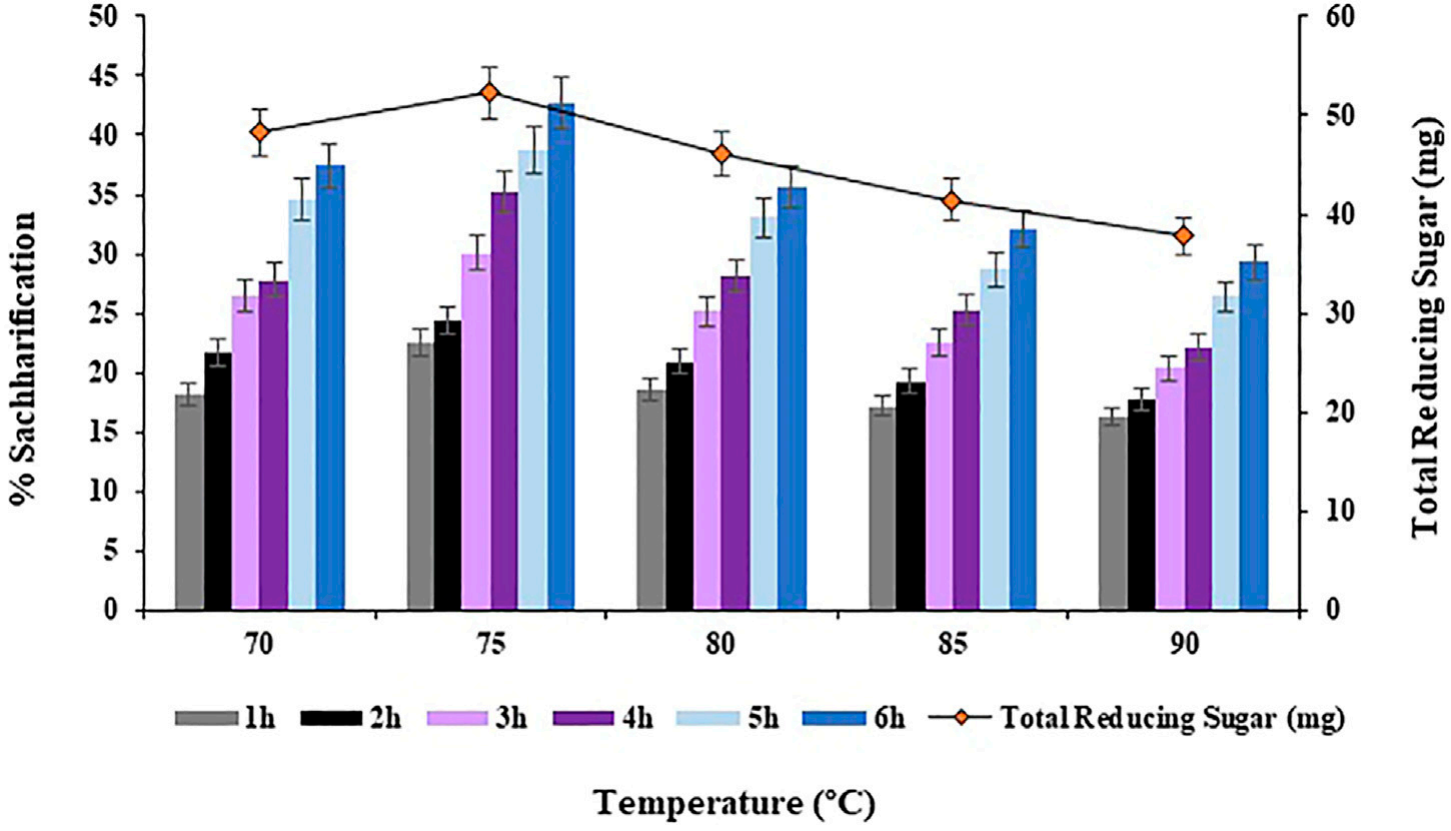
Effect of incubation temperature on saccharification of pre-treated halophyte *Atriplex crassifolia.*

Different incubation temperatures i.e. 70, 75, 80, 85°C and 90°C were analyzed for the bioconversion of substrate i.e., *Atriplex crassifolia* into saccharides. Maximum bioconversion of 42.7% (*p* < 0.05) was observed at 75°C after 6 h of incubating the reaction mixture that contained the cellulases and the pre-treated substrate i.e., *Atriplex crassifolia*, as shown in Figure 6. Increasing the temperature began to decrease the yield of saccharification, as shown in Figure 6. Hence, temperature of 75°C was optimized for the saccharification of pre-treated halophyte *Atriplex crassifolia*. Next saccharification experiments for the optimization of the remaining physicochemical parameters were conducted at temperature 75°C for a period of 6 h. This might be because enzymes work best at their optimum temperature. As the enzymes used in this study were cloned using thermophilic microorganisms, the maximum activity was exhibited at a high temperature. Further increase in temperature resulted in decreased saccharification, which might be due to enzyme denaturation (Saha et al., 2005). A temperature higher or lower compared to the optimum results in reduced transport across cell wall or enzyme denaturation (Dutt and Kumar 2014). Increasing both, the incubation time as well as temperature increases the saccharification rates, but upto a certain optimum point after which, the enzymes tend to lose their stability and activity, and the saccharification yields tend to decrease (Mondal et al., 2021). Majority of the hyperthermophilic microorganisms reported in literature do not efficiently cleave crystalline cellulose at temperatures <75 °C, because of the lack of carbohydrate-binding modules (Blumer-Schuette et al., 2008; Maki et al., 2009; Graham et al., 2011).

#### 3.3.4 Effect of substrate concentration on saccharification

**FIGURE 7:**
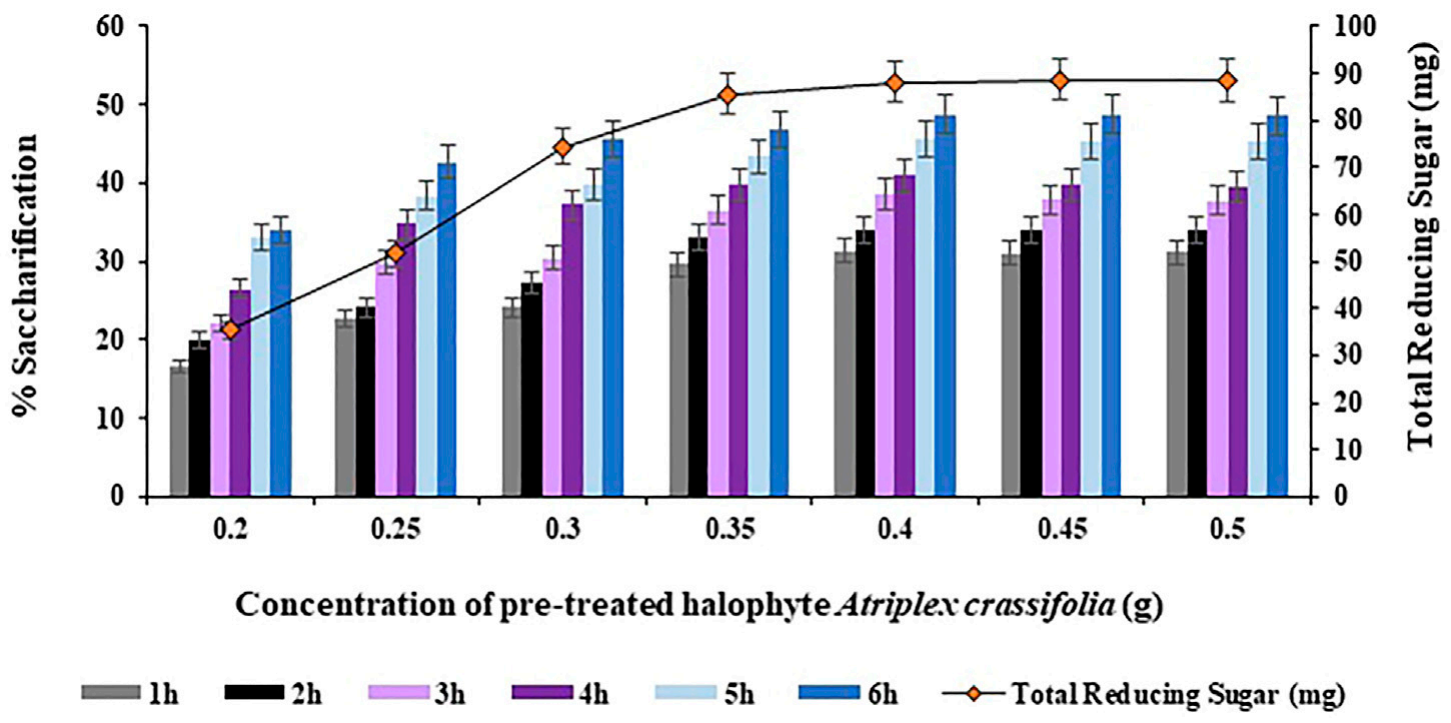
Effect of substrate i.e., pre-treated halophyte *Atriplex crassifolia* concentration on saccharification.

The concentration of substrate i.e., *Atriplex crassifolia* was varied i.e. 0.20, 0.25, 0.30, 0.35, 0.40, 0.45 and 0.50 g in order to achieve maximum saccharification. On comparing the saccharification yields achieved using the abovementioned concentrations of substrate, the highest saccharification yield of 48.8% (*p* < 0.05) was observed using 0.40 g of substrate at 75°C after 6 h of incubating the reaction mixture containing the cellulases and the pre-treated substrate i.e., *Atriplex crassifolia*, as depicted in Figure 7. Increasing the concentration of substrate while keeping other parameters constant, resulted in no additional increase in the yield of saccharification, as evident from Figure 7. Hence, substrate i.e., pre-treated halophyte *Atriplex crassifolia* concentration of 0.40 g was found optimum for maximum saccharification. Next saccharification experiments for the optimization of the remaining physicochemical parameters were conducted using 0.40 g of substrate i.e., pre-treated halophyte *Atriplex crassifolia* at temperature 75°C for a period of 6 h. One of the possibilities could be that all the pre-available enzymes ended up as enzyme-substrate complexes, and no additional free enzymes must be available to bind with the rest of the substrate, so as a result, exceeding the substrate concentration brings no change (Levine et al., 2011). Increased substrate concentration might also result in improper mixture ratio leading to lower yields because of improper interaction of enzymes with substrates. Besides that, substrate in a less quantity may become the limiting factor and prevent the bioconversion of substrate. The results were observed to be different compared to Bhagwat et al. (2016), who found 2 g of substrate to give best results for the bioconversion of *Parthenium hysterophorus*, by using a mixture of two enzymes i.e., cellulase and β-glucosidase. This is due to the reason that the optimum concentration of substrate differs depending on the substrate type and the enzymes in use.

#### 3.3.5 Effect of endo-1,4- β -glucanase, exo-1,4- β -glucanase and β -1,4-glucosidase concentration on saccharification

**FIGURE 8:**
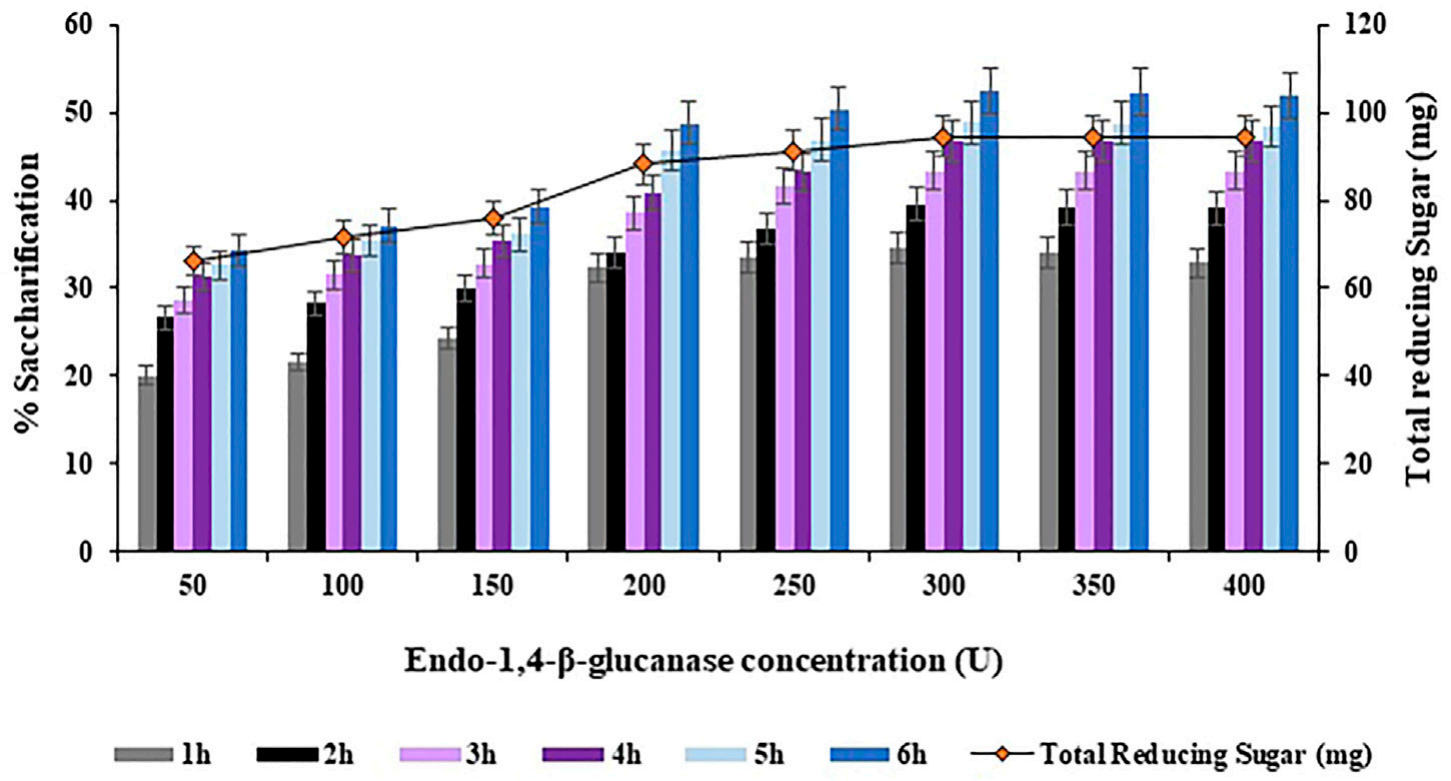
Effect of Endo-1,4- β -glucanase concentration on saccharification of pre-treated halophyte *Atriplex crassifolia.*

The concentration of Endo-1,4- β -glucanase was varied in a range of 50–400 U with an increment of 50 U in order to achieve maximum saccharification of the halophyte *Atriplex crassifolia*. On comparing the saccharification yields achieved using the abovementioned concentrations of Endo-1,4- β -glucanase, the highest saccharification yield of 52.4% (*p* < 0.05) was obtained with Endo-1,4- β -glucanase concentration of 300 U, using 0.40 g of substrate at 75°C after 6 h of incubation, as depicted in Figure 8. Increasing the concentration of Endo-1,4- β -glucanase while keeping other parameters constant, resulted in no additional increase in the yield of saccharification.

**FIGURE 9:**
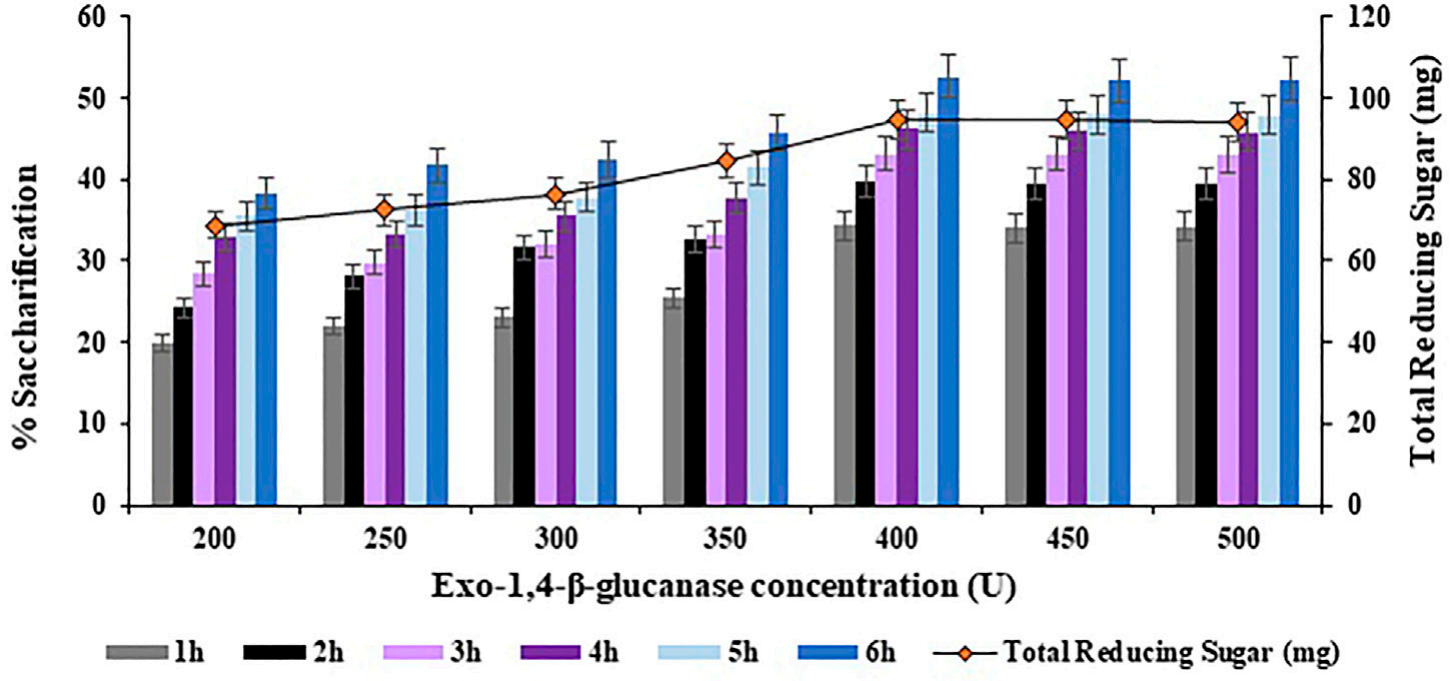
Effect of Exo-1,4- β -glucanase concentration on saccharification of pre-treated halophyte *Atriplex crassifolia.*

The concentration of Exo-1,4- β -glucanase was varied in a range of 200–500 U with an increment of 50 U in order to achieve maximum saccharification of the halophyte *Atriplex crassifolia*. On comparing the saccharification yields achieved using the abovementioned concentrations of Exo-1,4- β -glucanase, the highest saccharification yield i.e. 52.6% (*p* < 0.05) was obtained with Exo-1,4- β -glucanase concentration of 400 U, along with Endo-1,4- β -glucanase concentration of 300 U, using 0.40 g of substrate at 75°C after 6 h of incubation, as depicted in Figure 9. Increasing the concentration of Exo-1,4- β -glucanase while keeping other parameters constant, resulted in no additional increase in the yield of saccharification, as evident from Figure 9. Hence, Exo-1,4- β -glucanase concentration of 400 U was found optimum for maximum saccharification of the halophyte *Atriplex crassifolia*.

**FIGURE 10:**
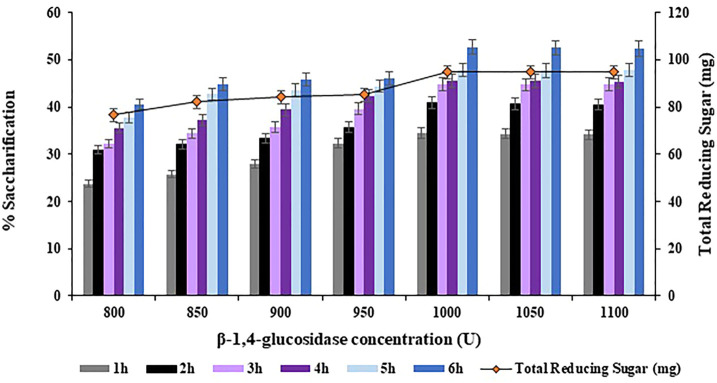
Effect of β -1,4-glucosidase concentration on saccharification of pre-treated halophyte *Atriplex crassifolia.*

The concentration of β -1,4-glucosidase was varied in a range of 800–1100 U with an increment of 50 U in order to achieve maximum saccharification of the halophyte *Atriplex crassifolia*. The highest saccharification yield of 52.7% (*p* < 0.05) was obtained with β -1,4-glucosidase concentration of 1000 U, along with Endo-1,4- β -glucanase concentration of 300 U, and Exo-1,4- β -glucanase concentration of 400 U, using 0.40 g of substrate at 75°C after 6 h of incubation, as depicted in Figure 10. Increasing the concentration of β -1,4-glucosidase while keeping other parameters constant, resulted in no additional increase in saccharification yield, as evident from Figure 10. Hence, Exo-1,4- β -glucanase concentration of 1000 U was found optimum for maximum saccharification of the halophyte *Atriplex crassifolia*.

Maximum saccharification of halophyte *Atriplex crassifolia* i.e. 52.7% was obtained with Endo-1,4- β -glucanase concentration of 300 U (Figure 8), Exo-1,4- β -glucanase concentration of 400 U (Figure 9) and with β -1,4-glucosidase concentration of 1000 U (Figure 10), when added simultaneously to conduct enzymatic hydrolysis, keeping other parameters as optimized above. Increasing the concentration of enzymes further resulted in constant saccharification yield, might be due to the onset of saturation point. As the time passes, the substrate would be converted into product, using up the enzymes’ active sites (Yang and Wyman, 2004). Hence, an optimum amount of each of the three enzymes is a pre-requisite to increase percentage saccharification. Enzyme loading is directly linked to the yield of reducing sugars, where higher enzyme loading yields greater quantities of reducing sugars, but upto a certain extent (Mota et al., 2021). Yu and Li (2015) obtained slightly different results, who utilized 200 U of an enzyme mixture sourced from *Gracilibacillus* sp. SK1, exhibiting a collective activity of endoglucanase, exoglucanase and β-glucosidase, for the saccharification of lignocellulosic biomas. The difference in the enzyme concentration might be because the optimum ratio of enzymes required to carry out hydrolysis may vary with each substrate and the source of enzymes being used (Chandel et al., 2010).

### 3.4 Bioethanol production

**FIGURE 11:**
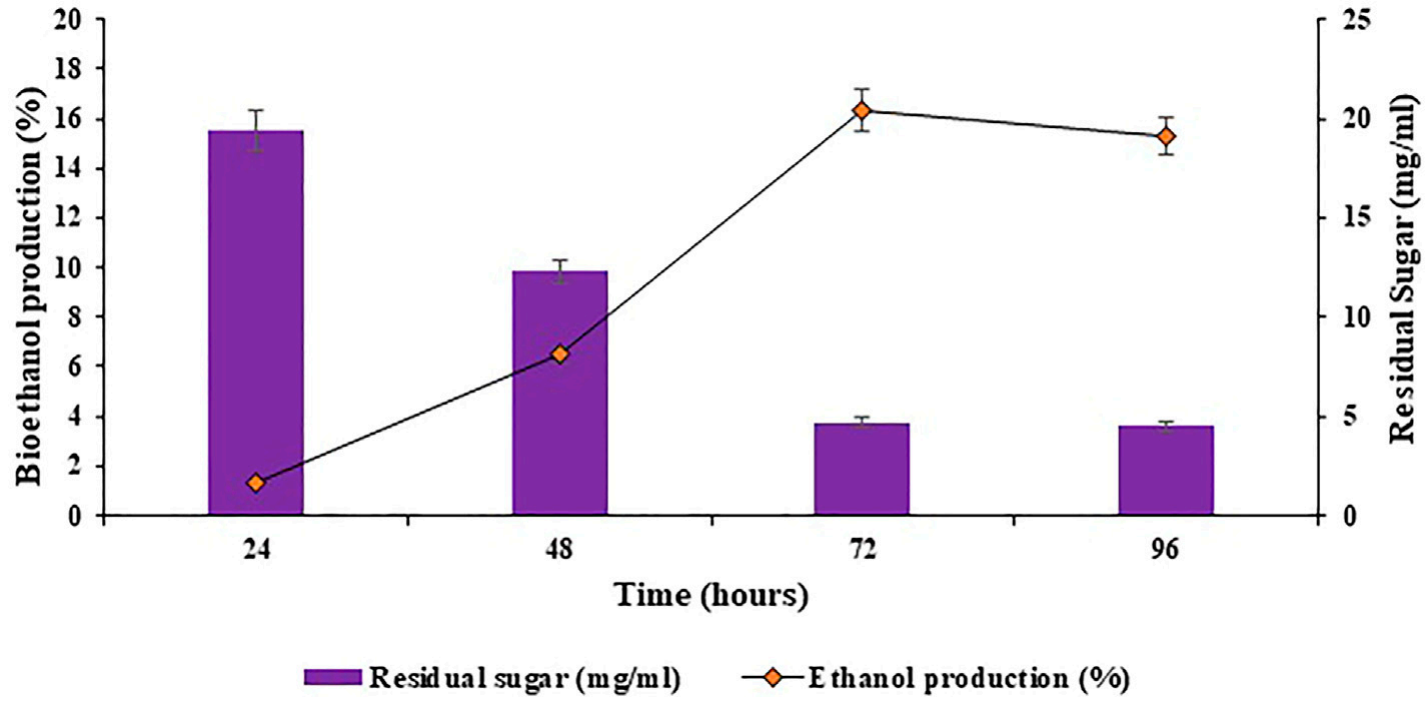
Production of bioethanol using the reducing sugar slurry obtained after optimization of physicochemical parameters for saccharification.

The slurry containing reducing sugars obtained after optimization of physicochemical parameters for saccharification was utilized in submerged fermentation, in replacement to glucose, for bioethanol production. The submerged fermentation for ethanol production was done in fermentation medium by making use of the reducing sugar slurry. The fermentation medium was inoculated with 2.5% of seed inoculum of *Saccharomyces cerevisiae,* under aseptic conditions. The inoculated reagent bottles were incubated at 30°C and 180 rpm for 96 h. The samples were harvested after regular intervals to estimate the ethanol content during fermentation. Maximum bioethanol production percentage observed was 16.33% at 72 h, as shown in Figure 11. Exceeding the incubation time further did not exceed the rate of bioethanol production, as evident in Figure 11. This might be because of the unavailability of enough reducing sugar to produce more bioethanol. Cotana et al. (2015) obtained comparable results for bioethanol production using *Phragmites australis* (common reed) as the feedstock, the highest yield of ethanol noted was 16.56 g ethanol/100 g of sample.

## 4 Conclusion and future recommendations

It can be concluded from the study that the halophyte *Atriplex crassifolia* owing to its high cellulosic content after pre-treatment using dilute acid method, yields substantial amount of reducing sugars and high saccharification rates when subjected to enzymatic hydrolysis using thermophilic cellulases, under optimized reaction conditions. Hence, the halophyte *Atriplex crassifolia* is a beneficial substrate that can be utilized to extract fermentable saccharides for bioethanol production.

As a means to enhance the overall yield and the techno-economic feasibility of the process, certain aspects must be taken into consideration:

1. Every component of the halophyte *Atriplex crassifolia* encompasses the ability to produce industrially important products. It will require additional research to discover its full potential.
2. The plantation of suitable species of halophytes with low lignin contents will enable easy enzymatic action on the main cellulosic structure, and result in improved saccharification yields, hence add to the techno-economic feasibility of the overall bioconversion process.
3. Research on more competent enzyme cocktails is needed with more powerful and compatible enzymes to enhance the efficiency of the bioconversion process.
4. Metabolic engineering can be applied to produce the enzymes required for pre-treatment, saccharification and fermentation from a single microorganism. This could reduce the process time; hence add to the cost-effectiveness of the process and ease of application.
5. Moreover, the most common pre-treatment procedures existent are not environmentally benign. Although different green processes have evolved for the pre-treatment of biomass that offer promising benefits, however, their wide-scale applicability at industrial level demands high capital cost. Therefore, extensive research is a requirement to develop the most applicable techniques for pre-treatment that ensure ease of applicability along with affordability.

## Data Availability

The original contributions presented in the study are included in the article/supplementary material, further inquiries can be directed to the corresponding author.
